# A Rare Location for Heterotopic Gastric Mucosa

**DOI:** 10.1097/PG9.0000000000000067

**Published:** 2021-04-12

**Authors:** Henedina Antunes, Sílvia Mota

**Affiliations:** From the * Pediatric Gastroenterology, Hepatology and Nutrition Unit, Hospital de Braga, Braga, Portugal; † Clinical Academic Center (2CA), Braga, Portugal; ‡ Life and Health Sciences Research Institute (ICVS), ICVS/3B’s-PT Government Associate Laboratory and School of Medicine, University of Minho, Braga, Portugal.

## INTRODUCTION

Heterotopic gastric mucosa (HGM) can be found anywhere in the gastrointestinal tract but is most commonly located in the proximal esophagus (1). It can be an asymptomatic finding in an esophagogastroduodenoscopy (EGD) performed for another reason or present with symptoms such as dysphagia, laryngospasm, hoarseness, globus throat sensation, and chronic cough (2).

### CASE REPORT

**Fig 1.**
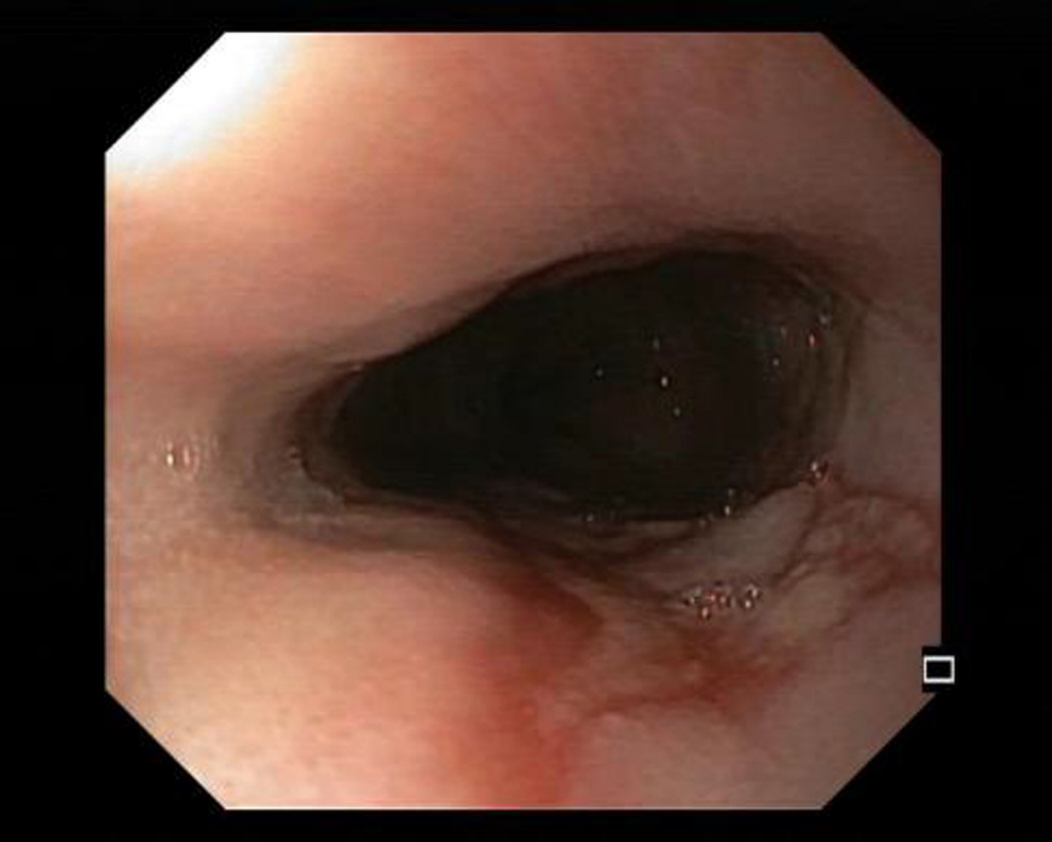
Middle esophagus with a vertical erosion.

**Fig 2.**
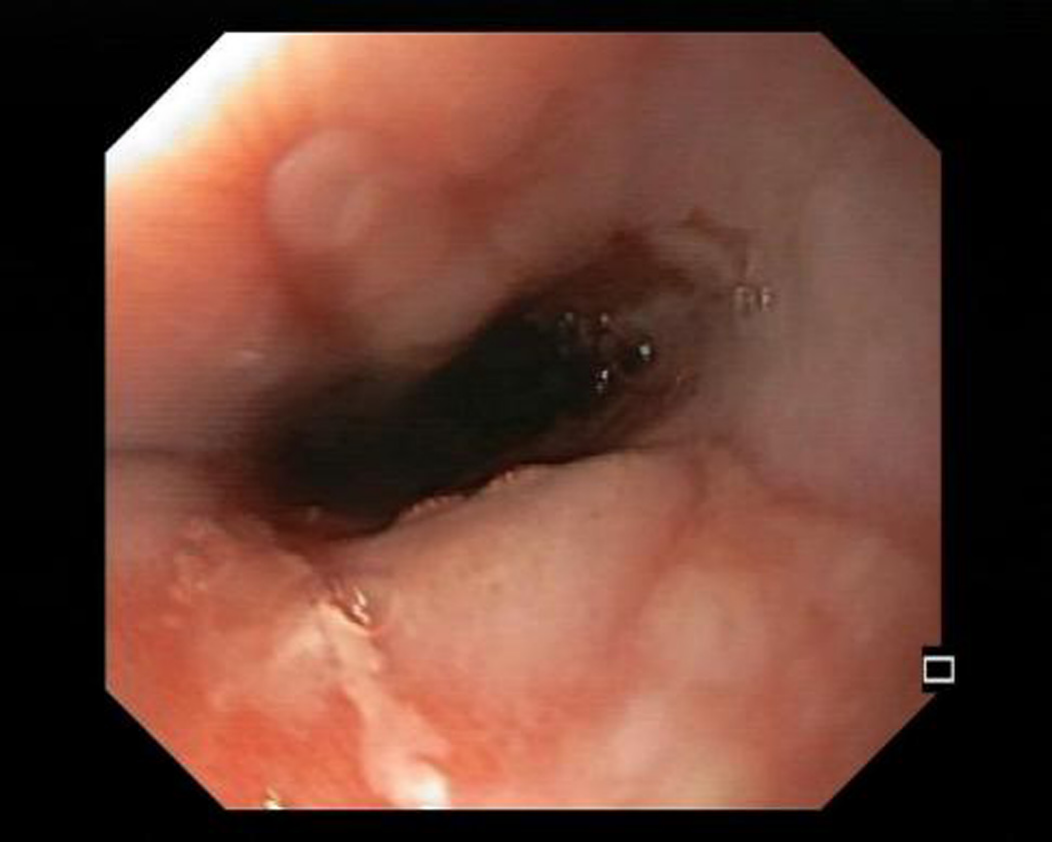
Distal esophagus with lesions consistent with esophagitis.

We describe a boy presenting in our institution at the age of 10-year old. He had history of gastroesophageal reflux disease (GERD) in the first year of life, treated with a proton pump inhibitor (PPI) and alpha-1 antitrypsin deficiency (AATD) (genotype Pi*ZZ), with cholestatic hepatitis treated with ursodeoxycholic acid. At the age of 6, he had two episodes of self-limited food impaction. An EGD was performed on the suspicion of eosinophilic esophagitis (EoE) but showed grade C esophagitis of middle third of the esophagus and EoE was histologically excluded. Treatment with PPI was initiated. On re-evaluation, 4 weeks after PPI suspension, he maintained esophagitis (grade B). PPI was continued on an on-and-off approach and two more EGDs were performed, showing esophagitis with superficial erosion of the esophageal mucosa. Normal range gastrin and pepsinogen I and II excluded gastric hypersecretion, and a normal esophageal transit exam confirmed the absence of hiatus hernia. Nevertheless, he was proposed for a Nissen fundoplication. By this time, he approached our institution. Another EGD was performed, revealing a pink inlet vertical patch of the middle and distal esophagus (Figs. 1 and 2), with a significant extent but sparing the gastroesophageal junction. Histology confirmed esophageal HGM.

### DISCUSSION

Epidemiologic data concerning HGM is scarce, with a suspected prevalence ranging from 0.1% to 10% regarding endoscopic detection (1,2). A pediatric endoscopic study found a prevalence of 6.3% (2); nevertheless HGM is suspected to be an underdiagnosed pathology (1,2). Because of its usual proximal location, HGM is easily missed during routine endoscopic procedures performed for other reasons, since the proximal esophagus is often only briefly examined (1,3). The diagnosis is also challenging in a distal location as it can be easily confused with GERD or Barrett’s esophagus (4,5). The origin of HGM has spanned several theories but the most widely accepted is that HGM is probably congenital, due to default epithelization process (1). As many cases are incidental findings, the decision of whom to treat is a difficult one. Von Rahden et al proposed a HGM clinicopathologic classification in five stages, with an expectant attitude or treatment, according to classification (1).

This case presents with unusual HGM location and the differential diagnosis with esophagitis was challenging. In most cases, HGM is found accidentally, an often without symptoms. Our patient had a symptomatic presentation, food impaction, but was extensively studied before a definitive diagnosis was established.

We did not find any other case reporting the association of AATD and esophageal HGM in the literature, so we can only assume that this was an incidental finding of the two pathologies in the same patient. However, since both are underdiagnosed pathologies, the association may be underrecognized (1,2,6).

The clinical diversity of HGM ranges from asymptomatic to severe consequences such as strictures, ulceration, bleeding, perforation, fistulae, and even concern about malignant progression to esophagus adenocarcinoma, although this is a rare, sporadic event (1,2). Since this patient presented with grade C esophagitis at a young age, he raises concern regarding evolution towards other complications.

According to the von Rahden et al classification (1), the patient has a HGM III and the initial proposed treatment may be acid suppression. Multidisciplinary discussion between pediatric gastroenterology and pediatric surgery led to the conclusion that medical treatment was best suited to the patient at this point, due to the risk of iatrogenic perforation with endoscopic treatment and the risk of post-surgical stenosis at the anastomosis site with surgical treatment. Another factor in favor of medical treatment was the longitudinal extent of the HGM, which makes it prone to needing esophageal reconstruction if surgery is attempted. PPI was instituted permanently, leading to complete resolution of symptoms, without invasive treatment, endoscopic, or surgical removal. As the complications of HGM are directly acid related, we expect that permanent PPI treatment will avoid them. Nevertheless, the patient and his parents were informed that, in the future, there may be a need to restructure this treatment plan, which could include endoscopic treatment—argon plasma coagulation, radiofrequency ablation, or endoscopic mucosectomy—or even partial esophagectomy with resection of the affected segment (7). Nissen fundoplication is not an alternative treatment option for HGM, since the esophageal lesion is a consequence of local acid production and not a complication of acid reflux.

There are no established clinical guidelines regarding the follow up of esophageal HGM (7). Clinical follow up will be maintained on a regular basis, with endoscopic reevaluation every 2 years. If the patient presents with symptoms again an unscheduled EGD will be performed to promptly detect complications and ascertain the need for additional intervention.

In conclusion, HGM is an underdiagnosis condition, easily missed in routine EGD and, as showcased here, easily misdiagnosed as a more common disease. Depending on classification, conservative treatment may be advised but close monitoring is essential to expeditious intervention in case of complications.

The parents are aware of this Case Report and have they given their consent.

## References

[1] von Rahden BH Stein HJ Becker K. Heterotopic gastric mucosa of the esophagus: literature-review and proposal of a clinicopathologic classification. Am J Gastroenterol. 2004; 99:543–551 15056100 10.1111/j.1572-0241.2004.04082.x

[2] Di Nardo G Cremon C Bertelli L. Esophageal inlet patch: an under-recognized cause of symptoms in children. J Pediatr. 2016; 176:99–104.e1 27318379 10.1016/j.jpeds.2016.05.059

[3] Chong VH. Clinical significance of heterotopic gastric mucosal patch of the proximal esophagus. World J Gastroenterol. 2013; 19:331–338 23372354 10.3748/wjg.v19.i3.331 PMC3554816

[4] Mungan Z. Is it barrett’s esophagus or gastric heterotopia? Case Rep Gastroenterol. 2014; 8:282–285 25408631 10.1159/000368301 PMC4224247

[5] Lupu VV Ignat A Paduraru G. Heterotopic gastric mucosa in the distal part of esophagus in a teenager: case report. Medicine (Baltimore). 2015; 94:e1722 26496283 10.1097/MD.0000000000001722 PMC4620775

[6] Stoller JK Sandhaus RA Turino G. Delay in diagnosis of alpha1-antitrypsin deficiency: a continuing problem. Chest. 2005; 128:1989–1994 16236846 10.1378/chest.128.4.1989

[7] Ciocalteu A Popa P Ionescu M. Issues and controversies in esophageal inlet patch. World J Gastroenterol. 2019; 25:4061–4073 31435164 10.3748/wjg.v25.i30.4061 PMC6700698

